# General Didactics and Instructional Design: eyes like twins A transatlantic dialogue about similarities and differences, about the past and the future of two sciences of learning and teaching

**DOI:** 10.1186/2193-1801-1-15

**Published:** 2012-08-17

**Authors:** Klaus Zierer, Norbert M Seel

**Affiliations:** 1 Carl von Ossietzky Universität Oldenburg, Ammerländer Heerstraße 114-118, Oldenburg, 26129 Germany; 2 Albert-Ludwigs-Universität Freiburg, Rempartstraße 11, 3, Freiburg, OG 79098 Germany

**Keywords:** General Didactics, Instructional design, Model building, Teaching and learning, Comparative education

## Abstract

Although General Didactics (GD) and Instructional Design (ID) have not shown many points of contact in the past, there are some obvious parellels from the perspective of their historical development. This will be examined in detail in this article. More specifically, we speak about model building, which has characterized General Didactics and Instructional Design for some decades. However, the models of General Didactics and Instructional Design are not problem-free with regard to the continuity and advancement of both disciplines. First, we will describe the historical roots of both disciplines and examine which elements of theory are of central importance. Second, we will try to answer the question of which kind of model building could be considered as predominant and what problems result from this predominance. In order to do this, we will describe empirical studies on the use of instructional models and discuss these studies from the perspective of the philosophy of science. Third, we will draw inferences for future processes of model building in order to prevent the same problems that happened in the past from happening again. Finally, we will discuss the issue of what General Didactics can learn from Instructional Design and vice versa.

## Introduction

Within the realm of school pedagogics, the so-called *General Didactics* (GD) has a significant value. Its central task consists in planning and organizing successful processes of students’ learning. According to Dolch’s (1967) seminal definition, *didactics is the science of learning and teaching in general*. It deals with learning in all possible forms and with teaching of all kinds at all levels – initially without any reference to the possible content of teaching. Dolch’s definition corresponds largely to approaches that are based on theories of learning and focus on the analyzing and planning teacher, who may refer to information about the design of class instruction provided by didactics. Furthermore, this broad definition of didactics also corresponds to *Instructional Design* (ID), considered as the American way of planning and organizing instruction (Seel and Hanke 2011). However, Dolch’s definition does not apply to approaches of didactics that consider the content of learning and its justification as the central part of education. Actually, these approaches are committed first of all to the choice and preparation of content to be taught and learned. In addition, the related decisions must take into account the preconditions of the students. In German education, these content-oriented approaches refer to the concept of *Bildung*, which means cultivation or *education of the cultivated mind* (Bruford 1975). They clearly belong to the field of humanities and can be considered as a separate path to be found especially in German education. Some advocates are Spranger, Nohl, Flitner, Weniger, Litt, and particularly Klafki, who has advanced this branch of didactics since the late 1950s. Klafki’s approach has since been transformed into *critical-constructive didactics* (CCD), which can be considered as the prevailing model of German didactics to this day. The centerpiece of Klafki’s former argumentation is the *primacy of didactics* (i.e., WHAT should be taught and learned), whereas the *methods* of teaching (i.e., HOW something should be taught and learned) are considered subordinate. More generally, Jank and Meyer (2002) have expressed the core of didactics by asking the odd question “*Who should learn what, from whom, when, with whom, where, how, with what and for which purpose?*” This question is addressed by various didactic approaches. Within the aforementioned approach of didactics oriented toward the education of the cultivated mind, a distinction can be made between learning-oriented didactics, systemic didactics, constructivist didactics, communicative didactics, and others. By 1989, Nicklis criticized the existence of dozens of didactics which emerged from 1930 to 1990 (cf. Nicklis 1989).

Some of these didactic approaches, particularly the learning-oriented and systemic didactics, correspond to a large extent to Instructional Design in the United States. Actually, the term Instructional Design refers to the systematic and professional provisions for education or training. Considerations regarding planned instruction have been made at least as long as there have been institutions for instruction and training. The term Instructional Design itself appeared for the first time in the USA in the mid 20^th^ century. From its very beginning, Instructional Design was closely related to instructional technology, which is generally defined as the systematic application of theoretically and practically established knowledge to the development of learning systems, for which the name “*Instructional Systems Development*” (ISD) is also used. Often the relationships between Instructional Design, Instructional Systems Development, and Instructional Technology (IT) are expressed by the formula “ ” In a general sense, Instructional Design is defined as the entire process of instructional planning and implementation. It refers to the principles and procedures by which instructional materials, lessons, and whole educational systems can be developed in a consistent and reliable fashion. The principles and procedures can be applied to guide designers to work more efficiently while producing more effective and appealing instruction suitable for a wide range of learning environments and educational settings. However, Instructional Design is also a field of theory and practice within the larger field of instructional technology. Accordingly, the term Instructional Design is also used to denote a scientific discipline that refers to theory building and research on instruction aiming at human resources development. Thus, instructional designers work in various settings of human resources development, such as corporations, the military, and government agencies, but also schools, colleges, and universities. Similarly to the field of General Didactics, an abundance of Instructional Design models have been constructed to guide instructional designers in their work, particularly within the realm of human resources development (cf. Rothwell and Kazanas 2008; Tennyson et al. 1997).

No field of scientific endeavor is immune to criticism. That holds true with regard to Instructional Design as well as to General Didactics. Since the 1990s, critics of traditional Instructional Design approaches have grown increasingly strident in their complaints about its theoretical and epistemological foundations as well as its real and perceived shortcomings. Similarly, with its focus on several predominant models and “schools,” General Didactics was isolated from the international research on instruction until the 1990s. Therefore, didactic concepts of smaller range were increasingly postulated in explicit reference to empirical research on instruction, and especially to the field of instructional design (Flechsig 1987; Schott 1991).

By referring to the song title “Eyes like twins” of Wilson Phillips, we argue that General Didactics and Instructional Design have some obvious parallels from the perspective of their historical development and thus many similarities. Both fields focus basically on a similar understanding of teaching as the *making of learning* (as formulated by Willmann 1906). Of course, there are also some important differences between General Didactics and Instructional Design – and this may be one reason that both disciplines have not met each other often during the past five decades: The focus of General Didactics, for instance, is on the content to be taught, whereas Instructional Design focuses more on the methods of teaching. Nevertheless, we believe that General Didactics and some approaches of Instructional Design, especially constructivist Instructional Design, share many features. Accordingly, we believe that the two fields could learn a lot from each other. We hope that this article contributes to an integration of General Didactics and Instructional Design in order to promote expertise in instructional planning.

### Landmarks in the history of General Didactics and Instructional Design

When scholars refer to the history of General Didactics and Instructional Design, it seems to be common practice to trace back both fields to ancient times by referring, for instance, to Plato and Aristotle (Schrock 1995) as well as to Cicero or Quintilian (Zierer and Saalfrank 2012). Furthermore, a reference is often made to the 13^th^-century philosopher St. Thomas Aquinas, who discussed the perception of teaching in terms of free will, as well as to Johann Amos Comenius, the great theologian and philosopher who used the term didactics as one of the first thinkers in his famous work *Didactica Magna* and explained some principles of instruction which are still important today. Historically seen, other educators, such as Pestalozzi, Herbart, and Schleiermacher, must be mentioned as well as later, at the turn of the 20^th^ century, John Dewey in the United States and many reform pedagogues in Europe who can be considered as founders of modern instructional science.

Because of their shared commitment to issues of the planning, implementation, and evaluation of instruction, General Didactics and Instructional Design clearly also share historical roots extending far back into the past. However, most models of modern day General Didactics and Instructional Design have their origin in educational science as it remerged after World War II and in several important historical events. At this time, General Didactics and Instructional Design met each other for the first time. Actually, it is interesting to see the parallels of the emergence of models in both fields on both sides of the Atlantic with regard to the same issues. Of course, the various models of didactics are based on different theories and conceptions concerning learning and instruction (Jank and Meyer 2002), but interestingly, the differences between didactic models are often bigger than those between didactic models and models of Instructional Design.

#### A brief history of General Didactics

It is not easy to find a starting point to define the beginning of General Didactics. As mentioned above, its roots go back to the ancient world, and Comenius was one of the first who used *Didaktik* (didactics) as a technical term. But for the history of General Didactics as a science, a landmark may be seen in the university reforms after World War II: General Didactics was established and became an academic discipline. A lot of professorships contain General Didactics in their title. At this point, General Didactics changed from a pre-scientific art of instruction (cf. Franz Xaver Eggersdorfer, Josef Esterhues, Franz Huber, Gustav Rose, Karl Stöcker, etc.) to a scientific discipline of learning and teaching (cf. Bönsch 2011). This change is connected to two didacticans: Wolfgang Klafki und Paul Heimann. Both tried to set General Didactics on a scientific fundament and published two important articles: In 1958 Klafki published “Didaktische Analyse als Kern der Unterrichtsvorbereitung” (Didactic analysis as the core of lesson preparation), and in 1962 Heimann published “Didaktik als Theorie und Lehre” (Didactics as theory and teaching). Both articles were the beginning of great discussions about General Didactics and especially about didactic models. Klafki developed his first ideas on didactic analysis in his “bildungstheoretische Didaktik” and finally in his already mentioned critical-constructive didactics. Heimann worked together with Otto and Schulz and developed the so-called “Berliner Modell,” which Schulz later expanded and reformed into the so-called “Hamburger Modell.” The “Berliner Modell” and the “Hamburger Modell” are now subsumed under learning-centered didactics (LCD) and teaching-centered didactics (TCD), respectively. Both theoretical lines – critical-constructive didactics and learning-centered didactics / teaching-centered didactics – can be seen as the central theories of General Didactics because they dictated the discussions about General Didactics for nearly forty years and are still *en vogue*. All other later developed theories and models of General Didactics are indebted to and influenced by both.

At the beginning of the discussion between Klafki and Heimann, the two didactic theories could be seen as extreme contradictions of each other: Klafki started his didactic thinking with “Bildung,” whereas Heimann took learning as his center. But soon both came together step by step. Klafki, for example, integrated evaluation into his theory as an important point, and Schulz added a critical perspective on the existing society. The results were the aforementioned critical-constructive didactics and “Hamburger Modell.”

Besides these prevalent models, there were a lot of other more or less important models. Kron counted over 40 in his textbook from 2008 (Kron 2008): cybernetic didactics, materialistic didactics, transcendental-critical didactics, evolutional-theoretical didactics, critical-communicative didactics, psychological didactics, etc. Some authors call these didactic theories the “forgotten” didactics, because in spite of plurality and diversity only critical-constructive didactics and the “Berlin” and “Hamburg” models succeeded in achieving classic status (cf. Zierer 2012). Nevertheless, they afford some interesting new ideas on learning and teaching.

The aforementioned discussion about didactic models was flanked in the 1960s and 1970s by curriculum studies – this was the first really influential transatlantic discussion on the field of General Didactics. Saul B. Robinsohn, former head of the Max-Planck Institute, criticized the state of the art of teaching and learning in schools and argued that a revision of the curriculum was necessary: According to his thesis, the curriculum was totally overfilled and not up to date. This criticism was aimed at General Didactics as well: He called General Didactics old-fashioned and heroically oriented and claimed that it does not question the content it tries to implement. Thus, a new understanding of learning and teaching, a new understanding of “Bildung,” and a new understanding of curriculum seemed to be necessary. The result of this transatlantic encounter was that General Didactics nearly died off. In the heyday of the curriculum discussion, General Didactics was nearly lost. But curriculum studies could not live up to its claims. And the only thing left was an idea that was still interesting but which had not been integrated and implemented successfully. However, there were again efforts to combine General Didactics and curriculum studies – some more successful than others (cf. Hopmann and Riquarts 1995).

After this period General Didactics came back and again attained the rank of one of the most important sciences in the context of learning and teaching, especially in the case of teacher education. Until PISA and Co., General Didactics had a nearly undisputed position. After the release of the first results of the PISA study in 2000, however, General Didactics met with increasing criticism in the public. Some authors held General Didactics to be as good as dead and started looking for possible candidates to take its place. This led to heightened importance for the empirically oriented sciences of teaching and learning, pedagogical psychology, and subject didactics. However, this discussion did not spell the end for General Didactics – quite the contrary: A lot of didacticans banded together and developed innovative and new perspectives on General Didactics that both drew on the former and traditional theories of General Didactics and took an empirical and international perspective. This period is still in progress.

#### A brief history of Instructional Design

The various essays about the history of Instructional Design (e.g., Leigh 1998; Reiser 2001; Schrock 1995) agree on the assumption that the beginnings of systematic planning of instruction date back to the 1920s. Across the following five decades different approaches of instruction emerged to respond to changing requirements of the American educational system. In the 1950s, three strong movements influenced the field of instructional planning: (a) Skinner’s theory of operant conditioning and reinforcement, (b) Bloom’s taxonomy of educational objectives for the cognitive domain (Bloom et al. 1956), and (c) the cybernetic approach of *systems theory*, which turned out to be most influential within the realm of instructional science (Seel and Hanke 2011).

These movements culminated in the “birth” of Instructional Design in the 1960s, when (Glaser introduced the term “instructional design” into the literature and Mager (1962) published his approach on the construction of instructional objectives that should be operationalized in measurable terms. A few years later, Gagné (1965) published *The Conditions of Learning* (Gagné 1985), which turned out to be the most influential contribution to the emergence of Instructional Design as a new scientific discipline. In accordance with the idea of a hierarchy of learning processes, Gagné identified nine events of instruction that provided a fundamental basis for an instructional theory “to propose a rationally based relationship between instructional events, their effects on learning processes, and the learning outcomes that are produced as a result of these processes” (Gagné 1985). On the basis of the nine events of instruction, Gagné and Briggs (1974) developed a prescriptive model of Instructional Design that describes how to create instruction for all domains of learning as well as how to determine the content to be taught. The Gagné-Briggs model has three phases: (1) determine objectives, (2) sequence, and (3) create the external events of learning. This model, in combination with systems theory, had strong effects on the development of other Instructional Design models, such as the well-known model of Dick and Carey (1978/2005) and the approaches by Merrill (1983), Reigeluth (1979), Smith and Ragan (1999), and many others (see, for an overview: Reigeluth 1983; Tennyson et al. 1997).

All these Instructional Design models agree on the assumption that learning can be classified in accordance with similar cognitive operations and processes (i.e., “internal conditions of learning”) and can be facilitated by similar instructional methods and strategies (i.e., “external conditions of learning”). However, the various models of Instructional Design initiated by Gagné do not provide a homogeneous class but rather focus on different components and strategies of Instructional Design. For example, Kaufman (1972) focused on the particular role of needs assessment and strategic planning at different levels of education. In Kaufman’s view, Instructional Design should not simply start with the formulation of instructional goals but rather with an analysis of those instructional objectives that are useful for anticipated audiences in particular learning environments. Needs analysis and strategic planning is still one of the most important areas of Instructional Design and Instructional Systems Development today.

Another important extension of Instructional Design models inspired by Gagné was the idea of automatizing the design of instruction and providing computer-based expert systems for this purpose (Tennyson 1994). However, this intended automating of Instructional Design by means of expert systems can be considered as failed in general. Nevertheless, starting in the 1960s, Instructional Design advanced to become one of the most prospering fields of education in general and resulted in a major “watershed” for the professionalism of instructional designers by the mid 1970s. For the first time, instructional designers became responsible contract partners for course development ahead of subject-matter experts. Actually, the 1970s saw a proliferation of Instructional Design models based on the core of systems theory and Gagné’s nine events of instruction, so that by 1980 more than 40 such models had been identified and included in a comparative analysis (Andrews and Goodson 1980). All these models described an expressly linear, systematic, and prescriptive approach to instructional design.

Along with the emergence of these Instructional Design models, but in sharp contrast to their behaviorist orientation, some psychologists, such as Ausubel, Bruner, Cronbach, Glaser, Wittrock, and others, realized in the late 1960s what is occasionally called the “cognitive revolution” of psychology (Bruner 1990). To a great extent inspired by Piaget’s epistemology, these cognitive approaches of learning and instruction increasingly replaced the former behaviorist approaches (Shuell 1986). This type of “new” learning theory was called “educational learning theory” (Bereiter 1990). Its introduction has also been associated with a strong tendency to investigate complex instructional matters and to focus on the development of “free learning environments” (Farnham-Diggory 1972) that aim at providing opportunities for reflective thinking. More recently, this field has experienced the influence of constructivist learning theory and a shift from teacher-controlled to learner-centered instruction (Reigeluth 1989). This movement which can be considered as the *third generation of Instructional Design/Technology*, led to the emergence of novel Instructional Design models based on constructivist principles (e.g., Hannafin et al. 1999; Jonassen 1999; Shambaugh and Magliaro 2001) and has, in turn, stirred a vigorous response from advocates of more traditional models (e.g., Dick 1996). This controversial discussion amongst scholars has been named the *constructivism-objectivism debate* and can be interpreted as the expression of an essential uncertainty of Instructional Design theorists concerning the epistemological foundations of Instructional Design. However, this debate contributed essentially to the self-conception of Instructional Design by assimilating and advancing theories from cognitive science and emerging communication technologies. Examples include: personalized system of instruction (Semb 1997), problem-based learning (Boud and Feletti 1997), open learning environments (Hannafin et al. 1999), and “constructivist learning environments” (Jonassen 1999).

As a result of a Delphi study aiming at the identification of trends that may influence Instructional Design in the future, Ritchie and Earnest (1999) point out that “with each iteration, we enhance our understanding of how to impact the performance of individuals and organizations” (p 35) by means of designed instruction. In general, the models of Instructional Design can be classified into three “generations” of Instructional Design. The first generation includes the development of procedural Instructional Design models (often illustrated as flow charts) and is greatly influenced by Gagné and disciples. The second generation of Instructional Design models contains approaches that can be understood as realizations of “educational engineering” and that aim at the automation of parts of the overall design process. The third generation, however, contains approaches which aim at the derivation of theoretically sound and research-based principles for the design of complex learning environments. The present applications of Instructional Design correspond largely to the first and third generation of Instructional Design.

### The core elements of General Didactics and Instructional Design

#### Traditional models of General Didactics – the big one

**Figure 1:**
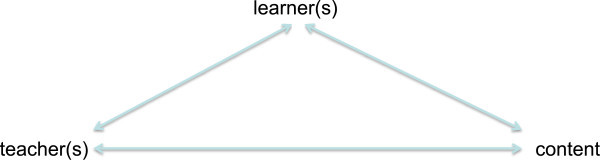
**The didactic triangle.**

Regarding the core elements of General Didactics there is one traditional model which every teacher has to know and every student has to learn: It is the so-called didactic triangle. In this case it is surprising that even very famous researchers do not know the roots of this approach (cf. Hudson and Meyer 2011), which already can be found in the ancient world: Aristotle developed the idea of a rhetorical triangle, composed of an orator, an audience, and a theme. This idea of a triangle was then taken up by Cicero in his work *De Oratore* and Quintilian in his work *Institutio Oratoria* and brought into a pedagogical and didactic context because both Cicero and Quintilian wrote about the education of an orator and thus expanded the rhetorical triangle into a didactic triangle. Its constituent parts are the learner(s), the teacher(s), and the content to be learned and taught. Additionally, it focuses on the different relationships between the constituent parts: the relationship between learner(s) and teacher(s), between learner(s) and content, and between teacher(s) and content (cf. Figure 1):

The triadic relation of learner-teacher-content is meant as an invitation to reduce the complexity of the didactic situation. The didactic triangle can be seen to offer tools that help sharpen the focus for planning and analyzing instruction (cf. Hudson and Meyer 2011). There is no doubt that the didactic triangle is so central for General Didactics that you can find it in all other didactic theories and models – for instance in critical-constructive didactics, the Berlin model, the Hamburg model, systemic didactics, or psychological didactics, to name only a few.

In the past decade there has been a lot of criticism of General Didactics in general, especially regarding the didactic triangle. The most important points of criticism are the following: First, it is reductionistic: Gruschka (2001) and Herzog (2010), for example, argue that the didactic triangle misses a lot of important aspects and is too simple for a holistic approach of teaching and learning. For example, it suggests that students can only learn when teachers teach them and it ignores the aspects of time, space, and interaction. Second, it is non-specific: Critical adherents of the idea argue that the idea of a triangle is also popular in rhetoric, communication science, media, and some other disciplines and contexts. So why should it be an extraordinary model for General Didactics? Third, it is not theoretically grounded: There is no theoretical background for the idea of a triangle. It is only a description of a situation, delivered over epochs and years without receiving an epistemological or scientific basis.

#### Traditional models of Instructional Design/Instructional Systems Development – The systematic approach

**Figure 2:**
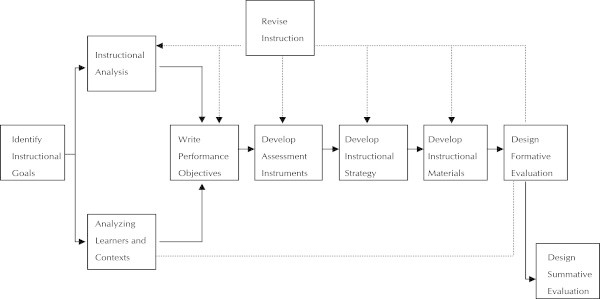
**The Dick and Carey model of Instructional Design.**

Many instructional designers first learn the process of designing instruction by studying one of the seven editions of Dick and Carey’s textbook *The Systematic Design of Instruction* (from 1978 to 2008). Dick (1996) asked in the course of the objectivism-constructivism debate whether the Dick and Carey model would survive the decade – the answer was provided by the fact that the 6th edition of the textbook was published in 2005 (cf. Figure 2):

The Dick and Carey model is probably the most influential model of Instructional Design and Instructional Systems Development. Thus, in accordance with this model, today’s Instructional Design and Instructional Systems Development models generally contain the following subtasks:

1. Conduct a needs analysis.
2. Determine if need can be solved by training.
3. Write learning objectives.
4. Conduct task analyses.
5. Identify the types of learning outcomes.
6. Assess trainees’ entry skills and characteristics.
7. Develop test items.
8. Select instructional strategies for training.
9. Select media formats for the training.
10. Pilot test instruction before completion.
11. Do follow up evaluation of training.

It should be noted that these are typical tasks and activities that will be found in most Instructional Design models; variations among professional practitioner models still exist. From a systemic point of view, these subtasks are interrelated, but they are currently no longer performed in a linear way or only a single time as in the past; rather, the current practice of Instructional Design is interactive and responsive to individual situations. Another commonly accepted improvement to the Dick and Carey model is the use of rapid prototyping (Tripp and Bichelmeyer 1990), which is a system development methodology based on building and using a model of a system for designing, implementing, testing, and installing the system.

Basically, Instructional Design is guided by a model of human performance improvement. Accordingly, Instructional Design/Instructional Systems Development can be applied at different levels of human resources development to address different clients, such as individuals, organizations (e.g., school settings), and the society (Kaufman et al. 1996). Actually, there are some examples of a successful application of Instructional Design to manage large-scale educational reforms in developmental countries (Morgan 1988,1989; Fretwell et al. 2001). In contrast to these effective applications, the current reforms of schooling – initiated by the PISA studies – are not guided by strategic planning, and the outcomes are thus not predictable.

Instructional Design/Instructional Systems Development models can be applied in all fields and situations that deal with education and training, including school settings, technical training, professional training, collaboration with human resource development professionals in industrial and professional organizations, higher education and university settings, and distance and life-long learning situations – using all kinds of media and telecommunications. In short, Instructional Design has matured into a broad profession with many connections to other professions and activities, including human resources and project management. It is especially noteworthy that practices of Instructional Design/Instructional Systems Development often involve a variety of experts, such as text designers, media designers, software programmers, subject-matter experts, and learning specialists. Teamwork is especially necessary when the development involves technology-based instruction since the required skills and expertise are likely to be distributed among a variety of specialists. However, when Instructional Design teams are involved, it is critical to have recognized standards and an established methodology as provided by Instructional Design/Instructional Systems Development models (Niegemann et al. 2008).

### The trouble with prevalent models of General Didactics and Instructional Design – Is there any trouble?

#### Trouble in General Didactics

**Figure 3:**
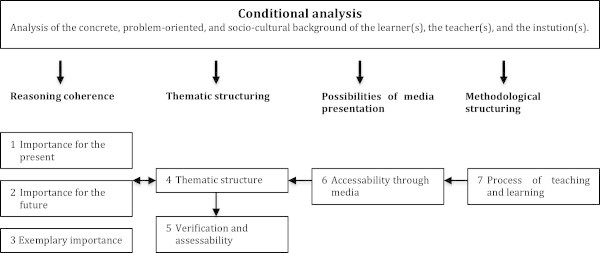
**The perspective schema for lesson planning of Klafki.**

As already mentioned, there are several prevalent models in General Didactics. In an analysis of modern textbooks of General Didactics, Zierer came to the result that two didactic models were always mentioned and described (cf. nal Design/Instructional Systems X2012): Critical-constructive didactics and teaching-centered didactics – both in their historical development. In the following, it seems important to describe both of these didactic models in their latest state of development:

At the center of critical-constructive didactics is the so-called “perspective schema for lesson planning,” an extension of Klafki’s “didactic analysis” (cf. Figure 3). It includes two related steps of analysis: The first is the conditional analysis, which focus on the concrete, problem-oriented, and socio-cultural background of the learner(s), the teacher(s), and the institution(s). The second is the didactic analysis, in which seven questions are answered: The first three have to do with reasoning coherence and ask whether the content to be learned is relevant for the present, the future, and as an example. The next two questions are concerned with the thematic structure of the content and aim at the goals and their verification and assessability. The next question focuses on the possibilities of media presentation, and the last question focuses on the methodological structure of the instruction. It is important to mention that these seven questions interact with one another and should thus not be seen as a rigid sequence that has to be gone through from one to seven.

**Figure 4:**
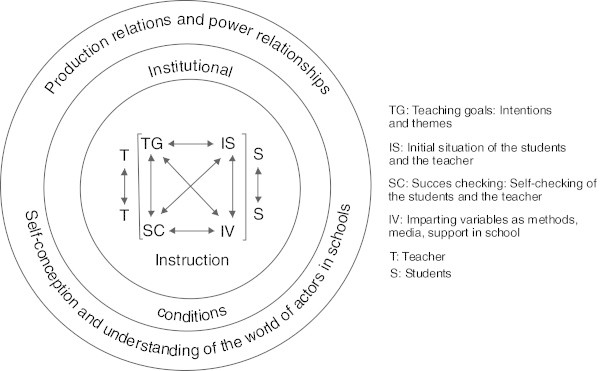
**The outline planning of an instructional unit of Schulz.**

At the center of teaching-centered didactics is the so-called “outline planning of an instructional unit,” which Schulz developed on the basis of the Berlin model (cf. Figure 4 and Arnold and Linder-Müller 2011). Although at a first sight it seems to still be a four-field planning schema like the Berlin model, there are several significant changes: Schulz subsumed intentions and themes under teaching goals and methods and media under imparting variables – both because of dependences and implications, as Schulz argued. Besides these two aspects, “success checking” and “initial situation of the students and the teachers” are the two other aspects of the four-field planning schema, which has to be seen in at least three interacting circumstances: first, the institutional conditions, which are important for every lesson plan; second, the production relations and power relationships, which show that all instruction is connected in a system view; and third, the self-conception and understanding of the world of actors in schools, which also influences the instruction.

Regarding the idea of lesson planning in general and the prevalent models in particular, there are several points of criticism worth mentioning. They focus on the following three aspects: lack of effectiveness, lack of usability, and lack of practicability.

First, lack of effectiveness: In the year 2000 when the first results of the PISA study were published there were great rumors both in the press and the scientific community. In Germany, for example, the results were poor and the German school system only ranked in the middle. Some critics see General Didactics as one of the reasons for the bad results, especially the dominant models. The argument was and still is that General Didactics is not fit for modern social and scientific developments but is still very popular in the concepts of teacher education. The critics argue furthermore that the dominant models every student has to know and every teacher has to learn are not as good as their founders and the members of the scientific community of General Didactics think. Thus, the critics argue that the dominant models lack effectiveness and bring the problem of General Didactics to a point: General Didactics is old-fashioned and not suitable for modern social and scientific development. Since that time, there have been a lot of discussions in Germany about the future of General Didactics and its place in the educational sciences (cf. Zierer 2012; Kiel and Zierer 2011; Terhart 2009; Arnold et al. 2009).

Second, lack of usability: This discussion about the right of existence of General Didactics renews some older studies about the usability of didactic models, such as the already mentioned Berlin and Hamburg models or the model of Wolfgang Klafki. In one of the first studies in this direction, Bromme analyzed the everyday planning procedures of teachers and came to the conclusion that teachers do not use the dominant models. Several other studies also came to the same result, also indicating that the dominant models have a lack of usability (cf. Bromme 1992). Although this is not the place to go into the problems of these studies, there is no doubt that these studies typify the discussions in Germany about the future of General Didactics and its place in the educational sciences.

Third, lack of practicability: As mentioned above, in the case of general didactics there are over 40 models. This shows that in the past decades the work and research on models has focused on differentiating between existing models rather than on a specific one. The result is that, first, there is not really any new knowledge in this field, and two major models are still dominant today: the Berlin and Hamburg models on the one hand and the model of Wolfgang Klafki on the other (cf. Seel 1999). Second, there are so many specialized theories that an overview of the field is not possible for a non-specialist and even for some experts. This development can be exemplified by a study by Thomas S. Kuhn (1962). Kuhn describes the history of science with the help of a paradigm, which he defines as generally accepted scientific benefits which are helpful for a specific time and period for solving major scientific problems. Paradigms are important for the scientific field because they structure the discourse and generate the basis and the state of the art of a discipline. What is important for this article is that a paradigm must not always and forever be a paradigm; rather, it can come to its end. Kuhn describes this process as follows: A paradigm comes to its end when the amount of knowledge about the main problem stagnates and a mere differentiation of existing theories takes the place of new developments. Kuhn describes this process as “excrescences.” The idea of building models in the fields of General Didactics (and Instructional Design as well) can be interpreted as a kind of paradigm: They are designed to help solve the main educational problem of theory and practice, to build a bridge between and overcome the specific differences between theory and practice. Therefore, models were developed for planning and analyzing processes of learning and teaching. But as already mentioned, the idea of building models seems to be heading down a dead-end street: None of the existing models is better than the others and none of the existing models can solve the defined problem satisfactorily. Thus, there are a lot of efforts and a lot of specialized and differentiated models. They could be interpreted as “excrescences,” which shows that General Didactics has a lack of practicability.

For many authors, these critics prepared the grave for General Didactics. Terhart, for example, argues regarding teaching-centered didactics that there is at the moment no sizable group of researchers who claim to work in this tradition and that it has been superseded by pedagogical psychology and the empirical science of learning and teaching. Against this background, General Didactics is as good as dead, and therefore some authors proposed several candidates that could take its place. Terhart suggested at least three potential candidates (cf. Terhart 2009):

First, subject didactics: A lot of empirical studies show that learning is very strongly connected to domains. Correspondingly, there are no general principles for learning. Thus, learning and teaching is always related to the different subjects, their content, and contexts. One idea for the future of General Didactics could be to subsume all existing findings of subject didactics. But this is a too narrow understanding of “general”: In spite of Aristotle, general is more than the sum of its parts.

Second, educational standards: After the presentation of the PISA results, politicians called for standards to enhance the school system and instructional quality, arguing that output instead of input and empirically verifiable competencies needed to take precedence over the nebulous concept of *Bildung*. This process is still in progress and is very influential. Nearly all curricula and syllabi were rewritten to take better account of competencies. But a closer look shows that this idea owes a lot to an idea from the past which failed: the curriculum discussion of the 1970s. As already mentioned, this reform focused on a stronger orientation toward empirical methods and evaluation. The danger of this perspective is clear and can already be seen in several developments: Teaching and learning were not seen as an answer for the subject and its development or for society and its needs. They were seen as answers for previous results of testing. And in some cases there is nothing else left than teaching students to take tests and the misunderstanding that the highest ranking means the best instruction.

Third, the so-called “Bildungsgangdidaktik”: This idea goes back to Herwig Blankertz. At its center is the vision that the *Bildung* of a single subject can be oriented toward a normative idea of *Bildung* in general. Thus, there is a very strong connection to constructivism because curriculum and syllabus must allow time and space for individual developments.

#### Trouble in Instructional Design

At the moment, there are two prevalent models of Instructional Design/Instructional Systems Development that refer to different levels of application: ADDIE at the macro-level and the Cognitive Load Theory at the micro-level of Instructional Design/Instructional Systems Development.

### ADDIE

In view of the vast amount of competing Instructional Design/Instructional Systems Development models, a comprehensive framework – called ADDIE – is widely used in Instructional Systems Development. Actually, ADDIE exists more as a label than as an Instructional Design model, and its origins are not clear at all. It seems that the ADDIE model is an umbrella term referring to a family of procedural Instructional Design models that share a common underlying structure of Instructional Systems Development. Although there is no original and elaborated ADDIE model (Molenda 2003), most theorists agree that ADDIE is an acronym that covers the major processes of the generic Instructional Systems Development process: **A**nalysis, **D**esign, **D**evelopment, **I**mplementation, and **E**valuation.

The phase of *analysis* contains, first of all, a needs analysis at different levels of application. Furthermore, this phase contains the analysis of objectives and performances as well as an analysis of the addressees and the tasks as well as a cost-benefit analysis of the planned instruction.

The *design* phase of ADDIE focuses on the design of a blueprint of the outcomes of instruction. Accordingly, the emphasis of this phase is on the design of a storyboard encompassing the entire structure of the instructional intervention or learning environment. At the center of design is the planning of instruction, and the necessary decisions refer to the orchestration of the external conditions for learning (e.g., methods of instruction, social interactions, media, and the organization of the environment).

The *development* phase of ADDIE contains the concrete realization of the decisions made in the design phase. More specifically, development concerns the production of the concrete learning material. The starting point of the development phase is a decision centered on the question “Buy it or make it.” In cases of doubt it is more efficient to work with pre-existing material than to produce it from scratch, and often it is more cost-effective to modify and adjust existing material to meet the requirements of a particular instructional task.

*Implementation* contains the concrete realization of planned instruction in a real setting, for instance an online setting. Characteristically, the implementation of instruction occurs under controlled conditions and critical examination. This is often closely associated with a formative evaluation during the development phase.

The *evaluation* phase of ADDIE refers to testing the success of instruction. The evaluation helps to measure the instructional impact on learners in terms of the pre-defined instructional objectives. Usually, a distinction is made between formative evaluation (i.e., a process-oriented evaluation of the development of instruction) and summative evaluation (as an instrument of quality management).

While it is perhaps the most popular framework of Instructional Systems Development, there are some weaknesses to the ADDIE model, especially with regard to the development of multimedia-based instruction. For instance, ADDIE tends to be inefficient because it is not sufficiently iterative. Its strict sequence of steps tends to work well for static content but may be restrictive when one is dealing with learning outcomes that do not have a predetermined end state (e.g., complex problem solving as an instructional goal). Another significant weakness is that there is no accommodation for dealing with faults or good ideas throughout the overall process of ADDIE. The designer must know all of the requirements before developing the learning material. Probably the biggest weakness, however, is that ADDIE is nothing other than a mere extrapolation of former Instructional Design models that does not take into account new approaches of instructional psychology. As a consequence, the ADDIE Model has been criticized as a “metaphor for the lack of clarity in the field of instructional design and technology” (Bichelmeyer 2004). Nevertheless, ADDIE can be considered as the preliminary final point of the development of Instructional Design/Instructional Systems Development models in the tradition of Gagné and disciples. ADDIE has initiated a number of spin-offs or variations, such as the approach of Decision Oriented Instructional Design (DO-ID) from Niegemann et al. (2008), which can be considered as the most detailed approach of Instructional Design/Instructional Systems Development for the time being.

The popularity of a theoretical approach in academia needs not imply popularity in practice. Actually, on the whole ADDIE is used only infrequently in the practice of Instructional Systems Development (cf., Magliaro and Shambaugh 2006). The application of ADDIE in practice is usually limited to some of its components and activities, e.g., the design phase. According to a study by Pieters (1995), this observation also holds true with regard to experts in the field of Instructional Systems Development. Although the participants in this study were trained in ADDIE as the most comprehensive model of Instructional Systems Development, most of them admitted that deviations and discrepancies did occur. For instance, the evaluation and implementation activities did not receive the amount of time and effort they deserve in ADDIE. This corresponds to the results of a survey study by Wedman and Tessmer (1993), who analyzed the design practice of 73 instructional designers developing training for business and industry. The purpose of the survey was to determine whether the instructional designers strictly followed the prescriptions of design models, such as the Dick and Carey model, or used the models selectively by focusing only on parts of them. The Dick and Carey model, which can be considered as the prototype of ADDIE, is based on three major assumptions: (a) All activities prescribed by the model must be completed; (b) each activity must be completed before one proceeds to the next activity; and (c) each activity must be completed at the same degree of precision. In view of the results of their survey, Wedman and Tessmer concluded that Instructional Systems Development models were not compatible with Instructional Design practice – professional instructional designers do not systematically perform all activities of a particular Instructional Systems Development model in practice but rather operate on a “*layers of necessity model*” by performing only activities considered necessary (see Tessmer and Wedman 1990). These observations were replicated in studies by Liu et al. (2002), Winer and Vásquez-Abad (1995), and others (see, for an overview of similar studies, Kenny et al. 2005). While instructional designers do make use of traditional Instructional Design models, they do not follow them in a rigid fashion but rather in accordance with necessity. They also engage in a wide variety of other tasks that are not reflected in Instructional Design models (Kenny et al. 2005).

Obviously, it is hard to realize ADDIE and related models of systematic design in practice due to the necessary time and effort. Nevertheless, one can still find the argumentation (e.g., Pieters 1995) that novice instructional designers should start with designing instruction by referring to traditional Instructional Design/Instructional Systems Development models such as the Dick and Carey model, that provide a structured generic problem for solving design problems. With increasing expertise instructional designers will change over to routines of Instructional Design/Instructional Systems Development that they develop in the course of time. Most probably, this also holds true with regard to another prevalent model of Instructional Design/Instructional Systems Development at the micro-level of planning: the Cognitive Load Theory.

### Cognitive Load Theory and the 4 C/ID model

Although the Cognitive Load Theory (CLT) emerged in the late 1980s, it can be traced back to the times of information science, programmed instruction, and related cybernetic principles of learning and educational design in the 1960s (Borko 1968; Lysaught and Williams 1963; Smith and Smith 1966). Along with Cognitive Load Theory in educational psychology, one can find similar conceptions of *information load* in the field of marketing and consumer research (e.g., Garbarino and Edell 1997; Hunter 2002), where it is argued that a large amount of information can lead to negative consequences such as poor choice and negative effects such as confusion or frustration.

Similarly, Berlyne (1971) has supported the idea of information overload, which may result from information containing too many elements, causing it to become too complex and difficult to process. Complexity increases with an increase in the number of necessary stages of processing required to perform a cognitive task. Task demands and complexity are mainly external, but both depend upon the learner’ goals and capabilities to perform a task. The *difficulty* of a task is clearly related to the necessary processing effort (i.e., the amount of cognitive resources to be activated) that is required from the individual to perform a given task, and is dependent upon context, state, cognitive capacity, and strategy or allocation of resources (Meijman and O'Hanlon 1984). In view of the various prior approaches that worked with the concept of information load, we are tempted to consider Cognitive Load Theory as “old wine in new bottles.” Indeed, Cognitive Load Theory is an extension of former theories that refers to a cognitive architecture corresponding to the stage model of information processing (Reed 2006). In this model, bottlenecks, restrictions in the flow and processing of information, occur at specific points, especially within the working memory with its limited capacity to process 7 ± 2 items simultaneously. Actually, Cognitive Load Theory describes learning in terms of information processing, involving a long-term memory which stores knowledge and skills on a permanent basis and a working memory which performs tasks associated temporarily. The premise of Cognitive Load Theory is that the quality of information processing will increase if emphasis is placed on the limitations of working memory. Accordingly, a basic assumption of Cognitive Load Theory is that working memory is limited to a storage capacity of 7 ± 2 items (Sweller 1988). This idea of a limited processing *capacity* can be found in several theories which can be traced back to Ebbinghaus (1885), Miller (1956), and Broadbent (1958) and operates with the concept of subjectively *experienced load,* which is defined as a demand placed upon human information processing. Accordingly, workload depends upon the interaction between an individual and a task, and the same task demands do not result in an equal level of workload for all individuals. Experienced cognitive load is not only task-specific; it is also person-specific (Norman and Bobrow 1975). Regularly, an overload may result if a learning task is too complex. Accordingly, the cognitive load approach deals with complexity using a single construct: *element interactivity*. If many elements interact, element interactivity is high; if few interact, element interactivity is low. Then the material is difficult to learn (Sweller and Chandler 1994).

On the basis of the assumption of element interactivity, a distinction is made between extraneous and intrinsic cognitive load. *Intrinsic load* occurs when information processing demands a lot of cognitive effort due to the complexity or difficulty of a task. *Extraneous load* occurs as a result of high interactivity among the elements in the learning material. For example, when studying a geometrical proof, students often need to combine information presented in both a diagram and a text. Because it requires mental effort to combine information presented in the two representations, a *split-attention* effect may occur, in which learners must continually shift their attention between the two representations. In addition, cognitive load may be affected by the effort needed for activating cognitive schemas. A basic assumption of Cognitive Load Theory is that the *activation of schemas* allows the limitations of working memory to be bypassed, resulting in automatic processing and thus minimizing the workload. This is called *germane cognitive load* and refers to the assumption that skilled performance develops through the construction of an increasing number of ever more complex and abstract schemas (Sweller 1994). Related with this argumentation is the idea of using *worked examples* as a substitute for schema activation. Worked examples are instructional devices that provide prototypical solutions for tasks to be accomplished (Atkinson et al. 2000). They resemble the concept of advance organizers in Ausubel’s assimilation theory of school learning (e.g., Ausubel and Robinson 1969).

In recent years, Cognitive Load Theory advanced to become one of the most influential theories of instructional design and resulted in an abundance of empirical studies on its various aspects. Basically, it aims at helping instructional designers to reduce the cognitive load caused by poorly designed learning materials. The goal of Cognitive Load Theory is to make material easy to learn and use. Proponents of Cognitive Load Theory (e.g., Errey et al. 2006) argue that learning is easier when an item can be understood in isolation. Learning becomes difficult when the number of items to be learned exceeds the capacity of working memory (i.e., 7 ± 2 items). Learning is also difficult when information is provided in different formats (e.g., text and graphics) and the learner has to integrate the different formats before a concept can be understood. The process of integration requires additional workload and makes the un-integrated material harder to learn. This is the “split attention effect.” According to Errey et al. (2006), the use of redundant material also results in additional workload; therefore, these authors argue that a single source of instruction yields superior performance. This argumentation seems, at first glance, to be plausible, but it contradicts constructivist approaches of instruction that focus on complex problem solving and discovery learning. Furthermore, it also contradicts results of communication and consumer research, for which Hunter (2002) states that empirical support for the existence of information overload has been ambiguous.

**Figure 5:**
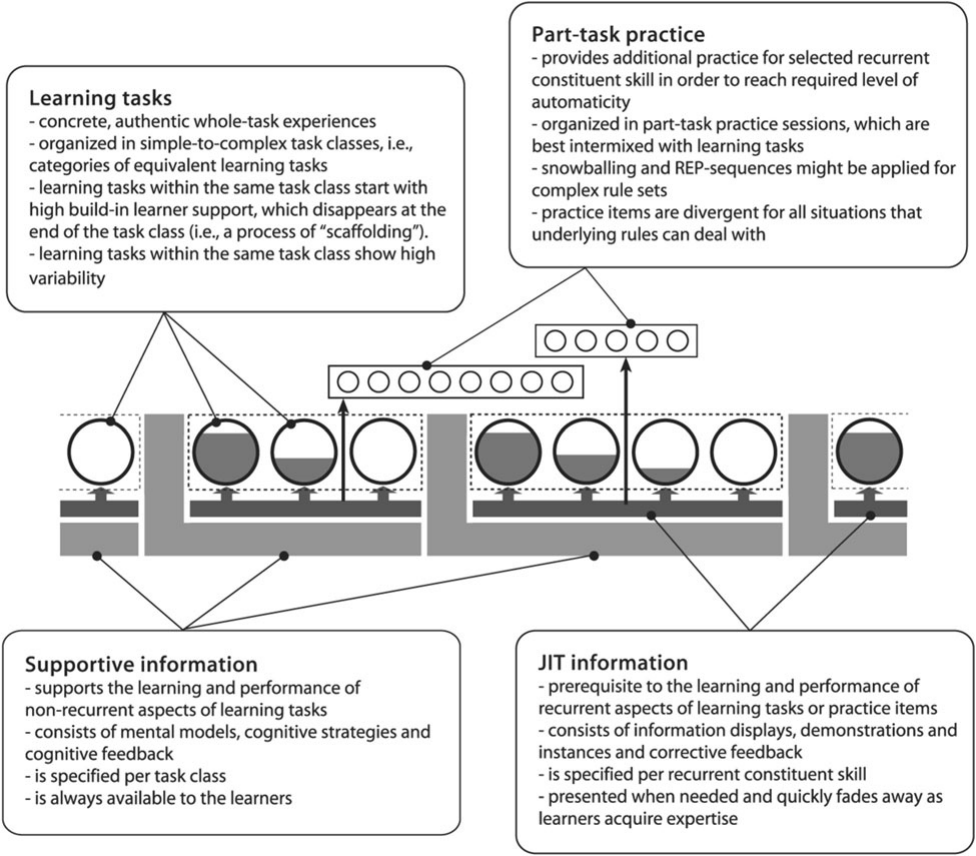
**The 4 C/ID model (van Merri**ënboer 1997**).**

However, inconsistent and contradictory results of empirical studies are quite normal and should not be overrated. What is more important with regard to Cognitive Load Theory research is that it has been typically performed in piecemeal fashion, in small-scale, specialized contexts, and without sufficient integration into comprehensive Instructional Design/Instructional Systems Development models. That conjures up the impression of not seeing the “forest for the trees.” For the time being, it seems that only van Merriënboer’s (1997) Four Component Instructional Design (4 C/ID) model integrates Cognitive Load Theory into a comprehensive framework of Instructional Design/Instructional Systems Development. In detail, the Four Component Instructional Design model considers authentic learning tasks as the driving force behind learning processes as well as the first component of an appropriate design of learning environments. The other components are *supportive information*, *procedural information* (recently also called “just-in-time information”), and *part-task practice*. The entire Four Component Instructional Design model is illustrated in Figure 5 (cf. Figure 5).

The Four Component Instructional Design model has attracted some research on its applicability in the practice of Instructional Design/Instructional Systems Development (e.g., Bastiaens and Martens 2007; Sarfo and Elen 2007; Sluijsmans et al. 2006). The results of these studies show that the Four Component Instructional Design model can be successfully applied in various fields of Instructional Systems Development. The Four Component Instructional Design model can be considered as a true innovation within the field of Instructional Design/Instructional Systems Development but it is mechanistic by nature and represents an objectivist view on instruction and learning. Also comparable with Cognitive Load Theory and ADDIE, the Four Component Instructional Design model argues that it is necessary and sufficient for effective instruction to design learning tasks and environments that initiate and guide learning processes. This point of view, however, stands in sharp contrast to constructivist and constructive approaches of Instructional Design, which operate on the basis of a completely different epistemology and understanding of learning and teaching. It is clearly a systematic addition to complex learning (van Merriënboer and Kirschner 2007) and it is problem-based, although not in the sense of typical problem-based learning models, as Merrill (2002a) states. At the core of the Four Component Instructional Design model is whole-task practice, “in which more and more complex versions of the whole complex cognitive skills are practiced. While learners practice simple to complex versions of a whole task, instructional methods that promote just-in-time information presentation are used to support the recurrent aspects of the whole task while, at the same line, instructional methods that promote elaboration are used to support the non-recurrent aspects of the task” (Merrill 2002b, p 8).

In the systematic approach, instruction is viewed as a process consisting of inputs, processes, and outputs. In this approach, the outputs or the outcomes of instruction are very precisely predefined and predetermined prior to the instruction. After the goals have been set, there are methods of teaching and learning to help the students achieve the desired outcomes. In contrast, the constructive approach of Instructional Design is based on epistemological and psychological aspects of constructivism and views Instructional Design as the preparation of resources and learning processes in order to facilitate students’ learning discussed in terms of the creation of meaning.

### Constructivist/constructive approaches of Instructional Design

As mentioned above, in addition to the development of the systematic approach of Instructional Design/Instructional Systems Development the constructivist approach of Instructional Design has emerged since the 1960s. In the systematic approach, which is based on behavioral and cognitive psychology, instruction is viewed as a process consisting of inputs, processes, and outputs. In this approach, the outputs or the outcomes of instruction are very precisely predefined and predetermined prior to the instruction. After the goals have been set, there are methods of teaching and learning to help the students achieve the desired outcomes. In contrast, the constructive approach of Instructional Design is based on epistemological and psychological aspects of constructivism and views Instructional Design as the preparation of resources and learning processes in order to facilitate students’ learning through the creation of meaning in their minds. There is no emphasis on predetermined design steps in the constructive approach. Rather, the emphasis is on the development and implementation of learning environments which provide the students with opportunities for inquiry and discovery learning.

In general, constructivism refers to different developments within various cultural sectors that are centered on the concept “construction” in theory development. In cognitive science, constructivism as a paradigm posits that learning is a constructive process of knowledge and mental representations (imagery, concepts, and mental models) of reality. Constructivist approaches emphasize some basic principles of learning, such as embedding learning in authentic contexts and social settings and providing opportunities for discovery learning and self-reflection. This worldview can be traced back to Vygotsky, Piaget, Dewey, and Bruner, who are usually considered as originators of constructivist learning theories.

In contrast to behaviorist approaches which were the source of systematic Instructional Design/Instructional Systems Development models, constructivism states that learning is an active, contextualized process of constructing knowledge and mental models rather than simply assimilating new information into prior knowledge. Learning is highly idiosyncratic, resulting in the fact that each learner will construct his or her own knowledge structures and mental representations. Naturally, the learner’s mind is not a *tabula rasa* but rather dependent on prior experiences and sociocultural factors. Constructivist approaches basically assume that all knowledge is constructed from the learner’s previous knowledge, regardless of how the learner is taught. Sometimes a misunderstanding regarding constructivist approaches of learning and instruction occurs when it is assumed that constructivist and inquiry-based instruction must fail due to its minimal guidance of the learning processes (Kirschner et al. 2006). This contention contradicts research on the effectiveness of guided discovery learning (e.g., Seel and Dinter 1995); it also confuses a particular theory of teaching with a theory of knowing (Dinter and Seel 1994).

An analysis of the literature shows that more than a dozen constructivist approaches of Instructional Design have been developed since the 1960s. They center on constructive, discovery, problem-based, model-based, experiential, and inquiry-based teaching aiming at providing the students with opportunities for reflective thinking (Stolurow 1973). Early constructivist approaches of instruction were developed in the 1960s and 1970s in the United States (e.g., Bruner 1966; Farnham-Diggory 1972) as well as in Europe (Aebli 1963). They drew strongly on Piaget’s view on education and emphasized the design of *free learning environments* in which students can develop their capabilities. Today, one can find numerous constructivist approaches of instruction, all of which are inspired by Piaget’s philosophy of education: (1) The *participatory design* model (Magliaro and Shambaugh 2006), especially the Recursive, Reflective, Design and Development (R2D2) model of Willis and Wright (2000); (2) Interpretation Construction (ICON) Design Model (Black and McClintock, 1995); (3) *generative learning and teaching* (Wittrock, 1974); (4) *discovery learning*, originally developed by Bruner (1966); (5) the *cognitive flexibility* approach (Spiro et al., 1991); (6) Computer Supported Intentional Learning Environments (CSILE) (Scardamalia et al. 1994); (7) *Mind Tools* (Jonassen 1996); (8) *anchored Instruction*, proposed by the Cognition and Technology Group at Vanderbilt (Pellegrino 2004); (9) the *cognitive apprenticeship* approach (e.g., Brown et al. 1989); (10) *case-based learning* and goal-based scenarios (Schank et al. 1993/94); (11) “*learning by design*” (Kafai and Ching 2004; Kolodner et al. 2004); (12) *model-centered learning and instruction* (Lehrer 2009; Seel 2003); (13) *problem-based learning* (Boud and Feletti 1997); (14) *project-oriented learning* (Thomas et al. 1999). It is not the place here to do justice to these approaches; most of them have been described extensively in the literature (see, for example, Reigeluth 1999; Seel and Dijkstra 2004; Ifenthaler et al. 2008). However, some points are worth making in this context.

First, the boundaries of these approaches are not sharp, but rather there are some substantial intersections. For example, “learning by design” can be considered as a variation of the participatory design model, in which students participate during the initial exploration and problem definition in order to define the problem and to focus their ideas on a solution and then evaluate proposed solutions during the design process. Such intersections also exist between model-centered instruction and problem-based instruction, and the “Interpretation Construction Design Model” operates with constructive design principles adapted from other approaches, such as cognitive apprenticeship, anchored instruction, and others (Black and Mc Clintock 1995).

Second, some of the aforementioned approaches only had a limited lifespan and must be considered outdated, such as, for example the cognitive flexibility approach, Computer Supported Intentional Learning Environments, mind tools, and generative. Because of the importance of models and modeling in mathematics and science (education), several model-centered approaches of learning and instruction, have been developed since the very beginning of the 1970s and are still promising today. Actually, two broad areas of model-centered instruction have been around for more than 40 years: One of them is based on pragmatics – and goes “beyond constructivism” (Lesh and Doerr 2003; Stachowiak 1973), while the other is largely inspired by the theory of mental models and goes “beyond pragmatism” (see, for more information, Seel 2009b).

Third, unlike the approach of systematic design and its emphasis on algorithms of planning and decision making, the aforementioned constructivist approaches consider instructional design as a *prototypical design activity* closely related to complex problem solving (Goel 1995). In contrast to the systematic Instructional Design, constructivist approaches operate with some principles of Instructional Design that can be derived from theory and research on learning and cognition. Constructivist approaches focus to a lesser extent on the procedures and methods of Instructional Design/Instructional Systems Development but much more on the creative design of learning environments aiming at effective learning and problem solving (Hanke et al. 2011). This can be illustrated by the examples of anchored instruction and model-centered instruction. Anchored instruction was based on theories of problem solving and, especially, on the role of analogical reasoning (Pellegrino 2004), whereas model-centered learning and instruction is based on mental model theory (Seel 2003).

In addition to the derivation of some guiding principles for the design of learning environments, constructivist approaches of Instructional Design preferably apply the methodology of rapid prototyping, where after a succinct statement of needs and objectives research and development are conducted as parallel processes that create prototypes, which are then tested and evolve into a final product. That’s why the design of instruction shares high-level characteristics with other prototypical design endeavors (Boling and Smith 2008). Instructional Design as a prototypical design activity is based on the assumption that Instructional Design/Instructional Systems Development is both a complex and ill-defined problem which can be characterized by some common features. According to Goel (1995), some characteristics are, for example, (1) a lack of information about a given initial state s_α_; a desired final state s_ω_; and a barrier which hinders the learner from solving the problem, i.e., from getting from s_α_ to s_ω_; (2) constraints on the task, either natural or artificial, do not constitute or define the task; (3) problems are large, complex, and sometimes dynamic; (4) it is difficult to break down a problem into distinct units; and others (Goel 1995, pp 85–87). In accordance with this conception, Instructional Design is considered as a matter of complex problem solving comparable with the areas of architecture, design engineering, graphic design, and other fields of design (Boling and Smith 2008). This corresponds to the Recursive, Reflective, Design and Development model of Willis and Wright (2000), who argue that instructional designers work on three aspects of the design process (definition, design, and dissemination) “in an intermittent and recursive pattern that is neither predictable nor prescribable. The focal points are, in essence, a convenient way of organizing our thoughts about the work” (p 5).

Fourth, constructivist approaches have promoted basic research in the area of Instructional Design/Instructional Systems Development. This is noteworthy because the quality of published research in the field of Instructional Design and instructional technology is generally poor, as Reeves (1997) and Stokes (1997) have stated. Actually, constructivist approaches of Instructional Design in general have been a strong motivation for empirical research on the effectiveness of designed learning environments. This can be easily demonstrated by referring to the research on anchored instruction and model-centered learning and instruction. Interestingly, this research has situated both anchored instruction and model-centered learning in the classroom, with a focus on observing the emergence of students’ qualitative models of phenomena to be explained. In general, this research provides really impressive examples of modeling activities in the classroom. This research on subject matter-oriented model-centered learning has been related often, but not exclusively, to qualitative research methods, such as collecting verbal data from think-aloud protocols, observational data, and videotape analyses. In addition, some researchers feel obliged to do design-based research and consider model-building in the classroom as a testing ground for *design experiments* (Seel 2009b). Design experiments aim at particular forms of educational interventions that create novel conditions for learning and instruction (Brown 1992; Lehrer et al. 2007).

Unlike the pragmatic approaches, research on model-centered instruction based on mental model theory seems to be more dedicated to experimental (and quasi-experimental) research and to the application of quantitative methods of data collection (Seel 2009a). As with the pragmatic line of research, research on mental models conducted during the past 30 years has resulted in a comprehensive and unique view on model-building activities under the condition of instruction. Most recently, the methodology of design experiments has been discussed as a heuristic for designing synthetic learning environments and simulation-based modeling (Seel 2009a). This corresponds to the fact that engineering a working (or learning) environment is the heart of a design experiment; according to Brown (1992), the working environment is nothing other than the instructional realization of a learning theory. However, at the moment the use of design experiments as a heuristic for designing synthetic learning environments and simulation-based modeling is still in its infancy and lacks empirical evidence.

This also holds true with regard to other new approaches of Instructional Design, such as the architectural approach of Gibbons (2012) or design as storytelling (Parrish 2006).

### A look into the future

#### The future of General Didactics

**Figure 6:**
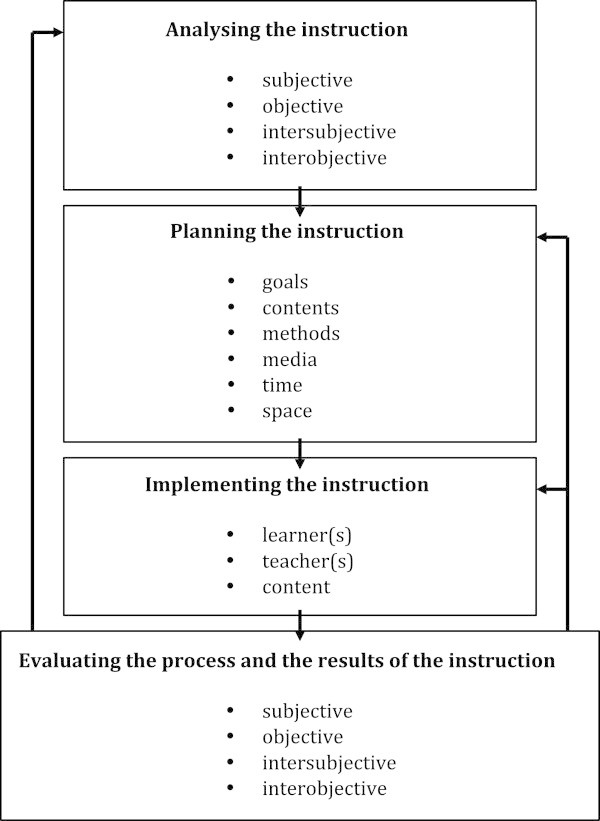
**Eclectic didactics.**

At the moment there is a discussion about the future of General Didactics (cf. Meyer and Meyer 2009; Kiel and Zierer 2011). For example, Meyer and Meyer title their article “There’s Life in the Old Dog Yet!,” and Kiel and Zierer have published an article titled “General Didactics Is Dead! Long Live General Didactics!” The reason for this can be found in the prevalent models, which seem to fail at their claim of being practical, effective, and useable. But nevertheless General Didactics is still alive, and at the moment there are two big interest groups regarding the future of General Didactics: The first one focuses on the many different didactic models and tries to integrate them and add new theoretical elements. This process might be compared with the position of ADDIE: In the context of Instructional Design, ADDIE can be seen as an umbrella term. An example of an approach of this kind in General Didactics is the so-called “eclectic didactics” of Zierer (cf. Zierer 2010 and Figure 6):

A first view shows that this approach tries to integrate the classic models of General Didactics as well as elements of Instructional Design. Thus, the instructional process is sequenced in four steps: analysis, planning, implementation, and (both formative and summative) evaluation, in which formative evaluation can influence the implementation and the planning and summative evaluation can influence the analysis. Here are a few comments on these four steps:

In the phase of analysis the so-called four-quadrants model by Ken Wilber is added (cf. Wilber 2002). Wilber developed his model in contrast to Jürgen Habermas’ three-world theory. It distinguishes between four major epistemological approaches: first, a subjective approach which aims at gathering information about the learner(s) and teacher(s) in the concrete situation of instruction, about their beliefs, hopes, wishes, etc.; second, an objective approach, the goal of which is to gather information about effects on teaching and learning regarding the concrete instructional process; third, an intersubjective approach which aims at gathering information about the social background of the concrete instructional process, like details on the social environment at the school, the curriculum, the fundamental concept of *Bildung*, and the overall social background; and fourth, an interobjective approach which aims at gathering information about the systemic influences on the concrete instructional situation. All of this information form the basis for planning the instruction, and therefore it is necessary to take a look at each of them. The phase of planning focuses on a didactic hexagon: goals, content, methods, media, time, and space. These are aspects that are present in nearly all didactic models, and they may be seen as the most important aspects in planning instruction. It is worthwhile to mention that these aspects all interact. This means that the fixed goals will influence the content, media, methods, time, and space and vice versa. In the phase of implementation it is of interest to keep an eye on the most important factors of instruction, i.e., learner(s), teacher(s), and content, as well as their changing und influential connections and relationships. In the phase of evaluation, the focus is, on the one hand, on a formative evaluation which concentrates on the concrete instructional process and offers the possibility to change aspects of planning and thus of implementation. On the other hand, the focus is on a summative evaluation, which concentrates on the final results of the concrete instruction. Thus, it delivers information, which will influence the analysis of later instruction, because the instruction will – hopefully – change learner(s) and their dispositions, thus making a new analysis necessary in the next instructional situation.

**Figure 7:**
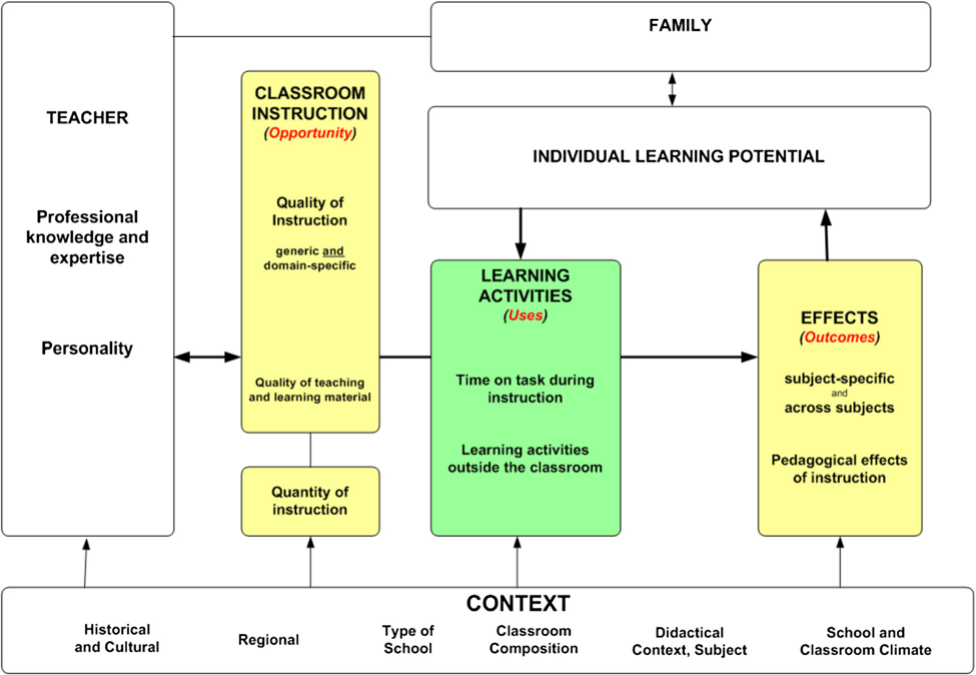
**Opportunity uses model of Helmke.**

The second interest group tries to develop new didactic models with empirical results from educational psychology or the empirical sciences of teaching and learning. In this context, the so-called opportunity uses model of Andreas Helmke seems to be the most popular one (cf. Figure 7 and Helmke 2010).

The core element of this model is a constructivist position: Instruction can only be seen as an opportunity. Its uses depend on a multiplicity of factors: the teacher, classroom instruction, individual learning potential, learning activities, family, the context, and effects. Thus, the aim of this model is to integrate these empirically grounded aspects of instructional quality into a comprehensive model.

A major challenge for the future of General Didactics could consist in the combination of these two approaches – first the integral approaches of classic didactic models and second the instructional models from pedagogical psychology. The advantage of the first consists in the fact that their theoretical foundations are very broad, while the advantage of the second is that their empirical foundations are very broad. Thus, the goal for future developments in General Didactics might very well lie in the challenge of integrating these two different approaches of research.

#### The future of Instructional Design/Instructional systems development

Instructional Design/Instructional Systems Development as a discipline experienced a substantial crisis in the 1990s – not only due to the objectivism-constructivism debate but also due to the criticism concerning the prevalence of models of systematic design in general, such as the Dick and Carey model and ADDIE. For example, Gordon and Zemke (2000) stated that Instructional Design in its current state could be considered “as good as dead” due to the fact that education and instruction were increasingly obliged to accommodate a diverse set of students who needed to learn and transfer complex cognitive skills to a varying set of complex real-world settings and contexts. Accordingly, the field of Instructional Design is seriously challenged by these new demands. In consequence, more powerful Instructional Design models that can deal with today’s scientific and societal demands are needed.

Chartier (1938) famous statement that “nothing is more dangerous than an idea when it is the only one we have” certainly once held true. However, when we reconsider the various trends of Instructional Design since 1990 we can state that theories and models of Instructional Design now come in different types and concern different worlds. In these different worlds, ideas about “how to help people learn better” lead to different answers to the two basic questions of Instructional Design: “What to teach?” and “How to teach it?” (van Merriënboer et al. 2002). Accordingly, we can observe that models of systematic Instructional Design/Instructional Systems Development still are broadly applied in academia because “there’s life in the old dog yet,” and most probably the Dick and Carey model as well as other models of systematic Instructional Systems Development will also survive this decade. Along with this, some of the constructivist approaches, such as model-centered learning and instruction, will also survive due to the fact that they are sufficiently capable of being integrated into school curricula (for instance those of math and science).

Aiming at both the further development of Instructional Design as a discipline in general and improvement of Instructional Design research, an international consortium of researchers organized by the International Center for Learning, Education, and Performance Systems (ICLEPS) of the Learning Systems Institute at Florida State University was established almost ten years ago. The cooperating partners and collaborators include faculty and researchers from the Open University of the Netherlands, the University of Freiburg (Germany), the University of Bergen (Norway), the University of Sydney, the University of New South Wales, SUNY-Albany, Syracuse University, the Learning Development Institute, the University of Central Florida, and the International Board of Standards for Training, Performance, and Instruction. Studies are conducted across all stages of development and levels of performance with the overarching goal being to create, test, and apply an integrated research foundation for the development of human learning and performance systems. Particular interest in learning in complex domains and the effective integration of advanced technologies are a hallmark of International Center for Learning, Education, and Performance Systems. Accordingly, International Center for Learning, Education, and Performance Systems builds on Cognitive Load Theory, mental model development, and Instructional Design theory to investigate: (a) human cognition and social processes that mediate learning and performance; (b) factors that influence human performance; (c) conditions that promote practical and effective learning; (d) methods to assess individual and team progress; and (d) methods to evaluate programmatic change and the diffusion of innovation. The focus is on the development of knowledge and skills in complex domains (i.e., those involving problem-solving and decision-making situations, for which there are often multiple acceptable or reasonable solutions) (see, for more information: http://www.ibstpi.org).

### What could/should General Didactics learn from Instructional Design and vice versa?

In view of the fact that General Didactics is concerned with education and training in school settings, whereas Instructional Design/Instructional Systems Development is more strongly related to human resources development, the question arises whether Instructional Design/Instructional Systems Development models share any feature with models of General Didactics. The answer is “yes” because there is hardly any difference between traditional models of systematic Instructional Design/Instructional Systems Development and the system-theoretical didactics of König and Riedel (1970) and its algorithms of instructional planning and decision-making. Actually, this particular didactic approach corresponds almost perfectly to the traditional models of systematic Instructional Design/Instructional Systems Development due to their common origins in systems theory. However, the system-theoretical model of didactics turned out to be very time consuming and inefficient with regard to the outcomes. Therefore, it was barely applied and is now completely out of date.

Furthermore, there is a basic correspondence between newer models of General Didactics and Instructional Design, for example between the aforementioned “eclectic didactics” of Zierer (2010) and Decision Oriented Instructional Design of Niegemann et al. (2008) due to their reference to processes of decision-making with regard to the major components of instructional planning: analyzing, planning, and evaluating on the one hand and intentions, contents, methods, and media of instruction on the other hand. This correspondence shows two important aspects and makes clear what General Didactics can learn from Instructional Design and vice versa: The first aspect is the theoretical orientation of General Didactics, which is often won by hermeneutical approaches. Almost all models of General Didactics have a very strong connection to a theoretical fundament. The best example for this is the critical-constructive didactics of Klafki, who developed his model on his concept of *Bildung*. The second aspect is the process orientation of Instructional Design, which is often won by empirical approaches and tries to integrate them, respectively. Almost all models of Instructional Design recur to ADDIE, which marks the steps of every instruction. Thus, a dialogue between General Didactics and Instructional Design will endeavor not only experience an opening regarding the theoretical concepts, it will endeavor an opening but also regarding the methods of educational research, too. And this is one and perhaps the most important point, which that General Didactics can learn from Instructional Design and vice versa: To be open-minded for the specific theories and approaches of the other discipline in its specific context and culture.

As long as General Didactics was primarily oriented toward the concept of *Bildung* as education of the cultivated mind, there was hardly any correspondence between this branch of didactics and Instructional Design models, which focused on the methods of instruction without ignoring the fact that learning takes place through the exercise of conceptual and procedural knowledge in the context of specific knowledge domains (Glaser 1984), whereas the didactic approach focused for a long time on the *primacy of didactics*, defined in terms of content to be taught. Klafki developed his model of didactics in the 1950s. Its goal was to establish a model for planning instruction centered on the content to be taught and learned. It aimed to examine content required by the curriculum with regard to its possible contribution to *cultivation*. At that time, Klafki’s didactic analysis focused on the present importance of any teachable content with regard to the students’ life, the expected importance of any content with regard to the future, the structure of the subject matter to be taught, its exemplary relevance, and its accessibility. Klafki’s schema is not a rigid and binding recipe but rather a guide for reflection and problematization. This also holds true with regard to critical-constructive didactics (CCD), which is a further development of Klafki’s former didactics and a sustainable response to the criticism concerning the former approach.

Critical-constructive didactics is *critical* in accordance with the *critical theory* of the Frankfurt School of philosophy (inspired by Hegel, Marx, and Freud and generally associated with Horkheimer, Adorno, and Habermas) (see, for more information: Gur-Ze’ev 2005; Tyson 2006). Klafki (1985) argues that didactics has to take into account that the reality of schools and societies constricts the attainability of objectives in multiple ways. However, didactics should not simply accept this but rather should work towards the elimination of barriers. Critical-constructive didactics is constructive because it does not settle for continuing to formulate proposals for instructional design within the realm of institutionally predetermined and curricular constraints, but rather didactics is designing something like a concrete utopia (Klafki 1985). The objective of teaching and instruction is to provide students with assistance in developing their capabilities of *self-determination and solidarity*. It aims to give students the means to justify and reflect on their actions. Additionally, it also focuses on developing the students’ emotionality and enabling them to have an active impact on society in accordance with reasonable objectives. This corresponds to Adorno’s (1969) concept of education aiming at maturity. Accordingly, students should acquire – with support from teaching – substantial *knowledge about the physical and social world* as well as the *ability to judge, to evaluate, and to act* in order to develop the ability of self-determined and independent learning and behavior. To achieve these objectives, learning must be meaningful and exploratory. Instruction should not aim at the mere reproduction and adoption of information but rather at providing opportunities for self-guided discovery learning. In accordance with Willmann’s (1906) precept of *teaching as the making of learning*, teaching has to support learning – not more and not less. This idea corresponds to the concept of free learning environments, which can be traced back to Piaget. Furthermore, critical-constructive didactics also corresponds to the *participatory design* model (Magliaro and Shambaugh 2006) and the approach of learning by design due to its emphasis on including the learners in the planning and design of instruction. Critical-constructive didactics postulates reasonable criticism and “instruction about instruction” as central components of classroom teaching with the aim of promoting social learning in the spirit of a democratic education. In accordance with the participatory design model, critical-constructive didactics demands the participation of students and teachers in consideration of their personalities, attitudes, and actions. Furthermore, the given social conditions as well as possible conflicts are considered to be central parts of instructional planning.

This overall program of critical-constructive didactics reads exactly like a constructivist approach of instructional planning. Indeed, both critical-constructive didactics and constructivist approaches of Instructional Design share the same “philosophy” of teaching and learning and are contrary to the objectivist approaches of Instructional Design and didactics.

At heart, it is only the critical component of critical-constructive didactics that makes this didactics different from the aforementioned constructivist approaches, due to the fact that critical theory is not established in North American education. Despite this, critical-constructive didactics is a perfect match for constructivist approaches of Instructional Design/Instructional Systems Development, especially the participatory model. We believe that merging the two approaches will result in a new quality of instructional planning and design – not only with regard to the necessary integration of critical theory into the field of Instructional Design/Instructional Systems Development but also with regard to the critical-constructive didactics' perspective schema of lesson planning. Accordingly, the analysis of the conditions of instruction should include the *context of justification* of the present, future, and exemplary importance of instructional objectives or projects. Furthermore, what Instructional Design/Instructional Systems Development could learn from critical-constructive didactics is a stronger emphasis on *thematical structuring*, i.e., the structure of topics as well as their confirmability and verifiability. Another important aspect concerns the accessibility and presentability (also through media) of the topics. Finally, the structure of teaching and learning should be understood as a variable concept of necessary and possible forms of organizing and realizing learning (including successive sequences) as well as providing appropriate learning aids. This comes along with the contention that teaching must be understood as social interaction and a medium of social learning processes.

Then it becomes clear that the differences between systematic Instructional Desing models and constructivist approaches of Instructional Design are much bigger than the differences between critical-constructive didactics (and related newer models like for example the, such as eclectic didactics) and constructivist Instructional Design models.
